# Prevalence and distribution of resistance and enterotoxins/enterotoxin-like genes in different clinical isolates of coagulase-negative *Staphylococcus*

**DOI:** 10.1186/s40001-020-00447-w

**Published:** 2020-10-12

**Authors:** Mona Nasaj, Zahra Saeidi, Hamed Tahmasebi, Sanaz Dehbashi, Mohammad Reza Arabestani

**Affiliations:** 1 Department of Microbiology, Faculty of Medicine, Hamadan University of Medical Sciences, Shahid Fahmideh Street, Park Mardome, Hamadan, IR Iran; 2 School of Medicine, Shahroud University of Medical Sciences, Shahroud, Iran; 3 Nutrition Research Center, Hamadan University of Medical Sciences, Hamadan, IR Iran

**Keywords:** Coagulase-negative *Staphylococcus*, Superantigenes, Antibiotic resistance

## Abstract

**Background:**

Coagulase-negative *staphylococcus* (CoNS) is considered to be the major reservoirs for genes facilitating the evolution of *S. aureus* as a successful pathogen. The present study aimed to determine the occurrence of genes conferring resistance to fluoroquinolone, determining of the prevalence of insertion sequence elements IS256, IS257 and different superantigens (SAgs) among CoNS isolates obtained from various clinical sources.

**Materials and methods:**

The current study conducted on a total of the 91 CoNS species recovered from clinical specimens in Hamadan hospitals in western Iran in 2017–2019. The antimicrobial susceptibility testing was performed using disk diffusion method and the presence of the IS256 and IS257, genes conferring resistance to fluoroquinolone and enterotoxins/enterotoxin-like encoding genes were investigated by polymerase chain reaction (PCR) method.

**Results:**

Among genes encoding classic enterotoxins, *sec* was the most frequent which was carried by 48.4% of the 91 isolates, followed by *seb* in 27.5% of the isolates. None of the CoNS isolates was found to be positive to enterotoxin-like encoding genes. In 11(12%) of all isolates that were phenotypically resistant to levofloxacin, 9 isolates (81.8%) were positive for *gyrB*, 8 isolates (72.7%) were positive for *gyrA*, 8 isolates (72.7%) harbored *grlB* and 7 isolates (63.6%) were found to carry *grlA*. The IS256 and IS257 were identified in 31.8% and 74.7% of the isolates, respectively. The results of statistical analysis showed a significant association between the occurrence of *staphylococcal* enterotoxins (SEs) encoding genes and antimicrobial resistance.

**Conclusion:**

Antimicrobial resistant determinants and SEs are co-present in clinical CoNS isolates that confer selective advantage for colonization and survival in hospital settings. The coexistence of insertion elements and antibiotic resistance indicate their role in pathogenesis and infectious diseases.

## Background

Coagulase-negative *staphylococci* (CoNS) serve as an important reservoir of antimicrobial resistance genes which can transmit between *staphylococcal* species or even other bacterial genera, but they have been implicated in rare cases of food poisoning [1, 2]. Among CoNS species, *S. epidermidis*, *S. hominis* and *S. haemolyticus* are often developed to be resistant to multiple antibiotics [3]. *Staphylococcal* exotoxins have been divided into three groups of *Staphylococcal* heat-stable enterotoxins (SEs), responsible for the pathogenesis of *Staphylococcal* food poisoning (SFP), exfoliative toxins (ETs) and toxic shock syndrome toxin 1 (TSST-1), causative agents of scalded skin syndrome and toxic shock syndrome, respectively [4]. Besides coagulase-positive *staphylococci* (CoPS), it is recognized that CoNS species are also capable of producing enterotoxins and associated with food poisoning outbreaks [5]. Several SEs are classified as SE-like (SEl) toxins due to the fact that they still have not been tested for emetic activity or lack the emetic properties [6]. Various types of classical SEs including SEA, SEB, SEC1, SEC2, SEC3, SED, SEE and SEH on the basis of their antigenicities are currently described [7, 8]. In addition to classical SEs, 16 new types of SEs (SEG, SEH, SEI, SER, SES, SET) and SEls (SElJ, SElK, SElL, SElM, SElN, SElO, SElP, SElQ, SElU and SElV) have been reported [6]. SEs are extracellular protein toxins with low molecular weight (26.900 - 29.600 KD) [8, 9]. They are heat resistant and retain their biological activity after treatment with majority of proteolytic enzymes [10]. SEs as well as toxic shock syndrome toxin-1(TSST-1) are belonging to the pyrogenic toxin superantigen family of exotoxins (PTSAgs) [11]. Rate of nosocomial infections (NIs) and antibiotics resistance is high in Iranian hospital. The increasing rate of NIs causes more antibiotics usage which leads to economic burden, and as a final result, the increases rates of morbidity and mortality. It had reported that the most prevalent blood stream and urinary tract infections pathogens were CoNS and *E. coli*, respectively. The highest resistance rate of CoNS and *E. coli* was against Penicillin (91.1%) and Nalidixic acid (57.7%). The most used invasive devices were intravenous canola (84.4%) and urinary indwelling catheter (81.7%), and patients which had invasive devices treatments showed higher rates of antibiotic resistance [12]. Antimicrobial drug resistance in CoNS is increasing which led to serious problems for therapeutic options [13]. CoNS acts as large reservoir of mobile genetic elements, which confer resistance to Β-lactams, aminoglycosides, quinolones, macrolides, and tetracyclines [13]. Fluoroquinolones are potent, broad-spectrum agents that were largely developed for the treatment of a wide range of infections due to Gram-positive and Gram-negative pathogenic bacteria [14]. The efficacy of more recent fluoroquinolones including levofloxacin, trovafloxacin, sparfloxacin, moxifloxacin, grepafloxacin and gatifloxacin have been confirmed for the treatment of various infections due to Gram-positive cocci [15]. The fluoroquinolones block activity of essential bacterial enzymes DNA gyrase and DNA topoisomerase IV, which involved in DNA replication [14]. GyrA and GyrB are the corresponding subunits of DNA gyrase, encoded by the *gyrA* and *gyrB* genes [16].Topoisomerase IV composed of ParC and ParE subunits (often referred as GrlA and GrlB in *Staphylococcus aureus*), encoded by *grlA* and *grlB* genes [14, 17]. Mechanisms of microbial resistance to fluoroquinolone include one or combination of five main bacterial mechanisms: alterations in the drug target, alteration in the cell permeability, quinolone-modifying enzymes, drug efflux pumps or gyrase protecting proteins [14, 15]. To our knowledge, this is the first report in literature describing the occurrence rate of SAgs genes among human CoNS isolates. Regarding to the impacting of antibiotic resistance and toxins in pathogenicity of CoNS isolates as well as due to association between the occurrence of insertion sequence elements IS256 and IS257 with antimicrobial drug resistance and pathogenesis, the current study aimed at assessing the prevalence of SEs and TSST-1 toxins, ISs, antibiotic resistance trends and the quinolone resistance determining regions (QRDRs) of *gyrA, gyrB, grlA* and *grlB* genes in CoNS thriving in various types of clinical specimens.

## Materials and methods

### Identification of CoNS isolates

A total of the 91 CoNS isolates were collected from various clinical specimens submitted to three teaching hospitals (including Beheshti, Besat, and Farshchian Hospitals) located in Hamedan, Iran, from September 2017 to November 2018. The origins of the isolates were as follows: blood, urine, catheters, and wounds. The included criteria were all patients with: (i) at least one positive blood culture for CoNS (ii) a central line in place for at least 48 h prior to a first positive blood culture (iii) who were treated with either linezolid, vancomycin or other antibiotics, methicillin-resistant and susceptible CoNS. The criteria for Exclusion were patients who transferred to other hospitals during treatment, as we could not assess outcomes such as recurrence or mortality. *Staphylococcus aureus*, Duplicate samples of same patients.

This study was approved by the ethics committee of the Hamadan University of Medical Sciences (Code No: IR.UMSHA.REC.1396.827).

### DNA extraction from isolates

The plasmid DNA Extraction Mini Kit (Favorgen, Taiwan) and Plasmid Extraction Kit (Sinaclon, Iran) were used for plasmid DNA extraction according to the manufacturer’s recommendations [18, 19]. CoNS Chromosomal DNA was extracted by boiling method [20]. Quality of extracted DNA was assessed by the Nanodrop ND–1000 (Nanodrop Technologies, Inc., Wilmington, DE, USA).

### Antibiotic susceptibility testing

The phenotypic antimicrobial susceptibility testing of various CoNS species was evaluated using a panel of 11 commercial antibiotic discs which belonged to various classes of antimicrobial agents. Disk agar diffusion (DAD) method was conducted according to the Clinical and Laboratory Standards Institute (CLSI) guidelines [21]. The antimicrobial agents used in current study were as follows: chloramphenicol (30 µg), cefoxitin (30 µg), clindamycin (2 µg), doxycycline (30 µg), erythromycin (15 µg), gentamicin (10 µg), levofloxacin (5 µg), novobiocin (5 µg), rifampicin (5 µg), trimethoprim-sulfamethoxazole (25 µg) and vancomycin (30 µg) (MAST, Merseyside, UK). *S. aureus* ATCC33591 was used as a quality control.

### Detection of SEs and TSST_1_ encoding genes

Multiplex PCR was used to analyze the following genes: *sea*, *seb*, *sec*, *sed*, *see*, *seg*, *seh*, *sei*, *selj*, *selm*, *seln*, *selo*, *selk*, *sell*, *selp*, *selq*, *selr*, *selu* and *tsst*. Multiplex PCR method was performed using five different sets of oligonucleotide primer mixtures (Set 1: *sea*, *seb*, *sec*; Set 2: *sed*, *see*, *seg*, *seh*; Set 3: *sei*, *selj*, *selm*, *seln*; Set4:, *selk*, *sell*, *selq*, *tsst*; Set 5: *selo*, *selp*, *selr*, *selu*). PCR amplifications were carried out in 25 µL volumes, containing 2 µL template DNA, 1 µL of each forward primer, 1 µL of each reverse primer, 7 µL (Sets 2, 3, 4, and 5) and 9 µL (Set 1) of sterile double distilled water and 8 µL of 2× Taq Premix-Master mix (Parstous Biotech Co, Iran). The primers used to amplify SEs and TSST-1 encoding genes are listed in Table 1 [22–24]. Each PCR amplification reaction was performed using a Bio-Rad thermocycler (Bio-Rad, USA) with the following cycles: initial denaturation for 5 min at 95 °C and then 35 cycles at 95 °C for 1 min (denaturation), 52 °C for 1 min (annealing) and 72 °C for 2 min (extension) and final extension was performed at 72 °C for 7 min. All PCR products were analyzed by electrophoresis for 50 min at 100 V through 1% agarose gel (Invitrogen). *S. aureus* reference strains were included in all reactions as positive control; ATCC 13565 (*sea*), ATCC 14458 (*seb*), ATCC 19095 (*sec*), ATCC 27664 (*see*), ATCC 51811 (*seh*), FRI 472 (*sed*, *seg*, *sei*, *selj*, *selm*, *seln*, *selo*, *selr*, *selu*).

**Table 1:** Primers used in this study

Gene targets	Primer sequences (5′ to 3′)	Amplicon size (bp)	References
*sea*	F: TTGGAAACGGTTAAAACGAA R: GAACCTTCCCATCAAAAACA	120	[22]
*seb*	F: TCGCATCAAACTGACAAACG R: GCAGGTACTCTATAAGTGCC	478	[22]
*sec*	F: GACATAAAAGCTAGGAATTT R: AAATCGGATTAACATTATCC	257	[22]
*sed*	F: CTAGTTTGGTAATATCTCCT R: TAATGCTATATCTTATAGGG	317	[22]
*see*	F: TAGATAAAGTTAAAACAAGC R: TAACTTACCGTGGACCCTTC	170	[22]
*seg*	F: TGCTATCGACACACTACAACC R: CCAGATTCAAATGCAGAACC	704	[22]
*seh*	F: CGAAAGCAGAAGATTTACACG R: GACCTTTACTTATTTCGCTGTC	495	[22]
*sei*	F: GACAACAAAACTGTCGAAACTG R: CCATATTCTTTGCCTTTACCAG	630	[22]
*selj*	F: CAGCGATAGCAAAAATGAAACA R: TCTAGCGGAACAACAGTTCTGA	426	[22]
*selm*	F: CCAATTGAAGACCACCAAAG R: CTTGTCCTGTTCCAGTATCA	517	[22]
*seln*	F: ATTGTTCTACATAGCTGCAA R: TTGAAAAAACTCTGCTCCCA	682	[22]
*selo*	F: AGTCAAGTGTAGACCCTATT R: TATGCTCCGAATGAGAATGA	534	[22]
*selk*	F: TAGGTGTCTCTAATAATGCCA R: TAGATATTCGTTAGTAGCTG	293	[23]
*sell*	F: TAACGGCGATGTAGGTCCAGG R: CATCTATTTCTTGTGCGGTAAC	383	[23]
*selp*	F: TGATTTATTAGTAGACCTTGG R: ATAACCAACCGAATCACCAG	396	[23]
*selr*	F: GGATAAAGCGGTAATAGCAG R: GTATTCCAAACACATCTAAC	166	[23]
*selu*	F: AATGGCTCTAAAATTGATGG R: ATTTGATTTCCATCATGCTC	215	[24]
*selq*	F: GGAAAATACACTTTATATTCACAGTTTCA R: ATTTATTCAGTTTTCTCATATGAAATCTC	539	[24]
*tsst*	F: AAGCCCTTTGTTGCTTGCG R: ATCGAACTTTGGCCCATACTTT	447	[23]

### Identification of genes conferring resistance to fluoroquinolone

The Quinolone resistance determining regions (QRDRs) of *gyrA*, *gyrB*, *grlA*, *grlB* genes were investigated by PCR amplification using specific primer sequences [25, 26]. Each 20 μL PCR reaction mixture contained: 10 µL of 2x Taq Premix-Master mix, 6 µL of sterile distilled water, 1 µL of forward primer, 1 µL of reverse primer and 2 µl of template DNA. PCR conditions for amplification of *grlA* and *grlB* genes: 95 °C, 4 min; 95 °C, 1 min; 48 °C (52 °C for *grlB*), 1 min; 72 °C, 60 s; 72 °C, 4 min; for *gyrA*: 95 °C, 1 min; 95 °C, 45 s; 54 °C, 60 s; 72 °C, 1 min; for *gyrB*: 94 °C, 2 min; 94 °C, 1 min; 48 °C, 60 s; 72 °C, 1 min; 72 °C, 5 min.

### Detection of insertion sequence elements IS256 and IS257 among CoNS isolates

Multiplex PCR assay was performed to identify insertion sequence elements IS256 and IS257, the appropriate oligonucleotide primers were selected as follows; for IS256 (762 bp), the 5′ primer AGTCCTTTTACGGTACAATG and the 3′ primer TGTGCGCATCAGAAATAACG; for IS257 (576 bp), the 5′ primer CTATCTAAGATATGCATTGAG and the 3′ primer TTAACTTGCTAGCATGATGC [17]. 25 µl of PCR mixture contained 2 µL of template DNA, 1 µl of each primer for IS256 and IS257, 10 µL of Master Mix, and 9 µL of sterile distilled water. The PCR conditions included an initial denaturation at 94 °C for 3 min, followed by amplification; 35 cycles at 94 °C for 1 min, 54 °C for 1 min, 72 °C for 2 min and 72 °C for 5 min.

### Statistical analysis

Cramer’s V, Phi- and Chi-square test were performed to assess of variables correlation. Phi and Cramer’s V have ranges from 0 to 1, where 1 indicates a significant association and 0 indicates no relationship. Interpretation of the Phi and Cramer’s V results; > 0; No or very weak, > 0.05 weak; > 0.10 moderate; > 0.15 strong; > 0.25 very strong. The Chi Square test was done by SPSS software version 20. P value < 0.05 was considered as statistically significant.

## Results

### Isolation and prevalence of CoNS isolates

Of the 91 clinical isolates of CoNS, 49 (53.8%), 37 (40.7%) and 5 (5.5%) isolates were recognized as *S. epidermidis, S. haemolyticus*, and *S. saprophyticus*, respectively. Isolates were recovered from blood 39 (42.9%), urine 33 (36.3%), catheter 13 (14.3%) and wound 6 (6.6%).

### Antimicrobial susceptibility testing

The results of antibiotic susceptibility testing of 91 CoNS isolates and the distribution rate of SEs and TSST-1 toxins among antibiotic resistant strains are demonstrated in Table 2. Among CoNS species, the highest resistance was observed for cefoxitin (53.8%), followed by trimethoprim-sulfamethoxazole (46.2%, n = 42) and none of the CoNS species was identified as being resistant to vancomycin. The highest percentage of genes that are involved in production of SEA, SEB, SEC, SEH, SEM, and TSST-1 toxins were significantly occurred among strains resistant to cefoxitin and trimethoprim-sulfamethoxazole with the frequencies of 100%, 100%; 52%, 48%; 63.6%, 50%; 50%, 50%; 80%, 60%; and 56.5%, 47.8%, respectively. In addition, among the phenotypically-resistant strains to cefoxitin and doxycycline, the distribution of SEE was significantly higher compared to strains resistant to other antibiotics (45.5% and 27.3%, respectively).

**Table 2:** Prevalence of superantigens among isolates with antibiotic resistance

Antimicrobial susceptibility patterns				Types of SAgs							
S	I	R	Antimicrobial agents	SEA (*n* = 2)	SEB (*n* = 25)	SEC (*n* = 44)	SED (*n* = 3)	SEE (*n* = 11)	SEH (*n* = 4)	SEM (*n* = 5)	TST_1_ (*n* = 23)
42 (46.2)	–	49 (53.8)	Fox	2	13	28	0	5	2	4	13
49 (53.8)	–	42 (46.2)	Sxt	2	12	22	1	2	2	3	11
62 (68.1)	3 (3.3)	26 (28.6)	Ery	1	8	13	1	1	0	2	5
73 (80.2)	–	18 (19.8)	Cli	0	4	7	0	2	1	0	2
64 (70.3)	4 (4.4)	23 (25.3)	Chl	1	5	10	1	1	1	1	4
88 (96.7)	–	3 (3.3)	Riph	0	3	1	0	1	1	0	0
80 (87.9)	–	11 (12.1)	Levo	0	4	3	1	2	0	0	2
100 (100)	–	0	Vanco	0	0	0	0	0	0	0	0
65 (71.4)	6 (6.6)	20 (22)	Gen	0	5	12	0	1	1	1	5
69 (75.8)	–	22 (24.2)	Dox	0	4	10	1	3	0	1	3
86 (94.5)	–	5 (5.5)	Novo	0	3	2	1	2	0	1	1

*Fox* cefoxitin, *Sxt* trimethoprim-sulfamethoxazole, *Ery* erythromycin, *Cli* clindamycin, *Chl* chloramphenicol, *Riph* rifampicin, *Levo* levofloxacin, *Van* vancomycin, *Gen* gentamicin, *Dox* doxycycline, *Novo* novobiocin

### Prevalence of Staphylococcal superantigen (SAg) genes among CoNS isolates

The distribution of SEs and TSST-1 encoding genes among *S. epidermidis*, *S. saprophyticus*, and *S. haemolyticus* strains and in various clinical specimens are shown in Table 3. Our findings demonstrated the high frequency of SEs genes among isolated CoNS from blood cultures, followed by urine, catheter, and wound, respectively. The *seg*, *sei*, *selj*, *selm*, *seln*, *selo*, *selk*, *sell*, *selp*, *selq*, *selr*, *selu* genes were not detected in any of the isolates. The *sec* gene was the most frequent, which was detected in 48.4% of the isolates, followed by *seb* in 27.5%, *tsst* in 25.3%, *see* in 12.1%, *sem* in 5.5%, *seh* in 4.4%, *sed* in 3.3%, sea in 2.2%. In the present study, enterotoxin-encoding genes were found in 39 (43%) isolates, from which 17(46%) belonged to *S. epidermidis*, 18(61%) were *S. haemolyticus* and 4(80%) *S. saprophyticus* strains were identified. Notably, the combinations of SEs genes were conserved in these strains. Amongst the positive isolates, 17 (53%) were positive for two genes, 12 (31%) were positive for three genes, and five (13%) harbored four genes. The most common combination of enterotoxin-encoding genes was *seb* and *sec*, which found in 18.7% of isolates, followed by *seb *+ *sec *+ *tsst* and *sec *+ *tsst* with frequencies of 15.6% and 12.5%, respectively.

**Table 3:** Prevalence of SEs and TSST_1_ encoding genes among various CoNS species and various clinical samples

Source					CoNS (*n* = 91)			
Catheter *n* (%)	Wound *n* (%)	Urine *n* (%)	Blood *n* (%)	Super antigenes	*S. epidermidis* (*n* = 49)	*S. haemolyticus* (*n* = 37)	*S. saprophyticus* (*n* = 5)	Total *n* (%)
0 (0)	0 (0)	1 (3)	1 (2.6)	SEA	0 (0)	2 (5.4)	0 (0)	2 (2.2)
4 (30.8)	1 (16.7)	12 (36.4)	8 (20.5)	SEB	14 (28.6)	8 (21.6)	3 (60)	25 (27.5)
6 (46.2)	3 (50)	15 (45.5)	20 (51.3)	SEC	29 (59.2)	13 (35.1)	2 (40)	44 (48.4)
0 (0)	0 (0)	2 (6.1)	1 (2.6)	SED	2 (4.1)	0 (0)	1 (20)	3 (3.3)
1 (7.7)	0 (0)	8 (24.2)	2 (5.1)	SEE	7 (14.3)	2 (5.4)	2 (40)	11 (12.1)
0 (0)	0 (0)	4 (12.1)	0 (0)	SEH	2 (4.1)	2 (5.4)	0 (0)	4 (4.4)
0 (0)	0 (0)	2 (6.1)	3 (7.7)	SEM	2 (4.1)	2 (5.4)	1 (20)	5 (5.5)
2 (15.4)	0 (0)	12 (36.4)	9 (23.1)	TST_1_	14 (28.6)	8 (21.6)	1 (20)	23 (25.3)

### The distribution of fluoroquinolone resistance genes among CoNS isolates

The distribution pattern of antibiotic resistance genes among CoNS isolates and in various clinical specimens are presented in Table 4. The blood cultures had the highest number of strains carrying genes responsible for inducing resistance to levofloxacin, followed by catheter and urine. The genes conferring resistance to levofloxacin were found in any of the strains obtained from wound. Among 11 of the phenotypically levofloxacin-resistant isolates, 9 isolates (81.8%) with *gyrB*, 8 isolates (72.7%) with *gyrA,* 8 isolates (72.7%) with *grlB* and 7 isolates (63.6%) with *grlA* were identified. The *gyrA* and *gyrB* genes were discovered as the most dominant genes inducing resistance to levofloxacin among *S. saprophyticus* strains and the *gyrB* and *grlB* genes were characterized with the highest frequency among *S. haemolyticus* strains.

**Table 4:** Prevalence of fluoroquinolone resistance genes among various CoNS species and various clinical samples

Source					CoNS (n = 91)			
Catheter *n* (%)	Wound *n* (%)	Urine *n* (%)	Blood *n* (%)	Resistance genes	*S. epidermidis* (*n* = 5)	*S. haemolyticus* (n = 4)	*S. saprophyticus* (*n* = 2)	Total n (%)
2 (100)	0 (0)	2 (100)	4 (57.1)	*gyrA*	4 (80)	2 (50)	2 (100)	8 (72.7)
2 (100)	0 (0)	2 (100)	5 (71.4)	*gyrB*	4(80)	3 (75)	2 (100)	9 (81.8)
2 (100)	0 (0)	0 (0)	5 (71.4)	*grlA*	4(80)	2 (50)	1 (50)	7 (63.6)
2 (100)	0 (0)	2 (100)	4 (57.1)	*grlB*	4(80)	3 (75)	1 (50)	8 (72.7)

### Prevalence of insertion sequence elements IS256 and IS257 among CoNS isolates

The distribution of IS256 and IS257 in the *S. epidermidis*, *S. saprophyticus*, and *S. haemolyticus* strains recovered from different sources are demonstrated in Table 5.

**Table 5:** Prevalence of insertion sequences IS256 and IS257 among CoNS isolates and various clinical samples

Source					CoNS (*n* = 91)			
Wound *n* (%)	Catheter *n* (%)	Urine *n* (%)	Blood *n* (%)	Types of IS	*S. epidermidis* (*n* = 49)	*S. haemolyticus* (*n* = 37)	*S. saprophyticus* (*n* = 5)	Total *n* (%)
2 (6.9)	2 (6.9)	12 (41.4)	13 (44.8)	IS256	12 (41.4)	17 (58.6)	0 (0)	29 (31.8)
3 (4.4)	11 (16.2)	24 (35.3)	30 (44.1)	IS257	37(54.4)	26 (38.2)	5 (7.4)	68 (74.7)

### Statistical analysis

Considering the Antimicrobial susceptibility patterns, it was found a significant association between novobiocin resistance and the occurrence of *sed* and *see* genes (*P* = 0.03, *φ* = 0.22 and *P* = 0.04, *φ* = 0.21, respectively), gentamicin resistance and the presence of *sec* gene (*P* = 0.01, *φ* = 0.32) as well as between resistance to rifampicin and trimethoprim-sulfamethoxazole and the incidence of *seh* and *see* genes responsible for production the SEH and SEE enterotoxins was observed in current study (*P* = 0.01, *φ* = 0.26 and *P* = 0.04, *φ* = 0.21, respectively). Among strains carrying insertion sequence elements found a significant association between the presence of IS256 and resistance to gentamicin as well as the occurrence of IS257 and resistance to cefoxitin (*P* = 0.04, *φ* = 0.25 and P = 0.004, *φ* = 0.30, respectively). In the current survey was also found meaningful relationship between the incidence of IS256 and production of SEA (*P* = 0.04, *φ* = 0.22).

## Discussion

This study provides the first report of the frequency of SEs and TSST-1 encoding genes among human CoNS isolates in Iran. Some investigators have reported lack of enterotoxin genes associated with human and veterinary CoNS isolates [27, 28]. The *sec* gene was the most common classic enterotoxin-encoding gene among all enterotoxin genes, which was detected in 48.4% of the isolates obtained from wound cultures. The frequency followed by *seb* and *see*, harbored by 27% and 12% of the isolates, respectively. While the *sea, sed, seh* and *sem* genes were rarely found (2.2%, 3.2%, 4.4% and 5.4%, respectively). The low prevalence of some SEs genes including *sem* in this study could be due to the theory that indicated enterotoxin-like (SEl) toxin genes are more abundant among commensal strains as compared to pathogenic ones [29]. Other studies also showed significantly higher rates of *sec* in CoNS isolated from coalho cheese, goat milk, industry meat and newborns, which ranged from 46.8% to 78% [5, 30–32]. Andrade et al. (2019) researched the occurrence of *sea*, *seb*, *sec*, *sed*, *see*, *seg*, *seh*, *sei* and *sej* genes in strains of *Staphylococcus* coagulase-positive (CoPS) and negative isolated from coalho cheese, and detected the presence of the following genes: *seh* (53.2%) in CoPS strains and *sec* (46.8%) in CoNS strains [30]. Lyra et al. (2013), also investigated SEs-encoding genes (*sea*, *sec*, *sed*, *see*, *seg*, *seh*, *sei*) in 44 strains of CoNS isolated from goat milk and found the presence of the *sec* in 55.6% of the strains and *sea* gene was found only in one strain [5]. These results indicate that the occurrence of the SEs genes isolated from coalho cheese and goat milk were not very diverse. Pinheiro et al. reported the presence of *sea*, *seb*, *sec*, *sed, see*, *seg*, *seh* and *sei* genes among *S. epidermidis* and S*. haemolyticus* isolated from blood cultures, while *sei*, *seg* and *sea* were the most frequent genes in both species [33]. It can relate to the fact that nosocomial isolates may be better equipped with virulence factors obtained by facilitated transfer through selective pressure. This is in contrast with other similar works which have been indicated the occurrence of *sea* gene in the higher frequency compared to other enterotoxin genes. Veras et al. evaluated the potential of CoNS and CoPS strains associated with outbreaks of *staphylococcal* food poisoning in Brazil and reported that enterotoxin genes were observed amongst 70% of the CoNS isolates. 38% found to harbor *sea*, 29% amplified only *seb*, and the concomitant presence of the *sea* and *seb* genes were reported in 24% of the isolates. Genes for *sec* and *sed* (either alone or concomitantly) were found infrequently [34]. In another study, the *sea* and *seb* genes have also described as the most common SEs gene among the *staphylococcal* species causing bovine mastitis [35]. These differences between studies may be related to geographic origin of the isolates, number and genetic structure of each isolate. In the current study, all of the strains producing of the enterotoxin SEA were positive for both *sea* and *seb*. The concomitant presence of these two genes in the same bacterium is explained by the fact that they occupy the same chromosome locus [36]. 17(46%) and 11 (61%) of toxigenic *S. epidermidis* and *S. haemolyticus* strains showed two or more the SEs genes in association that *seb *+ *sec* were detected as the most frequent. Other studies have also reported the concomitant presence of SEs genes in these organisms. Cunha et al. (2006) revealed the simultaneous presence of the *seb* and *sec* genes in 11%, *sea/seb* and *sea/seb/sec* in 5% of the CoNS obtained from newborns hospitalized in the Neonatal Unit of the Hospital of the Botucatu Medical School [32]. In another work, the concomitant incidence *sea* and *seb* genes have observed in 20% of the *S. epidermidis* and *S. haemolyticus* strains recovered from blood cultures [33]. The *tsst* gene was identified in 25% of CoNS species including 28.6% of *S. epidermidis*, 21.6% of *S. haemolyticus* and 20% of *S. saprophyticus* strains. In contrary with our work, other studies have found *tsst* gene in any of CoNS species isolated from newborns and cows with bovine mastitis [32, 37]. But this gene has been detected in 87% of *S. aureus* isolated from wound and blood cultures and in 15.5% of *S. aureus* obtained from bovine mastitis milk [38, 39]. Our findings demonstrated a high percentage of toxigenic strains among *S. epidermidis* (75.5%, *n* = 37), followed by *S. haemolyticus* (48.6%, *n* = 18). This is in accordance with other findings (Pinheiro et al. and Cunha et al.), which *S. epidermidis* was described as the CONS with the highest potential to produce of enterotoxins among identified isolates [33, 40]. The current study indicated a higher incidence of SEs genes among strains recovered from positive blood cultures(42.3%), so that 55% and 38% of all *S. haemolyticus* and *S. epidermidis* strains contained at least one enterotoxin gene, predominating followed by urine (37.2%), catheter (13.5%) and wound (6.7%) sources. This was in agreement with a similar survey carried out by Pinheiro et al. who exhibited a high prevalence of SEs genes in isolated *S. epidermidis* and *S. haemolyticus* strains from blood cultures with frequencies of 95.3% and 79.8%, respectively [33]. This is likely due to the fact that genes encoding some virulence factors were up-regulated in human blood over time. The coordinated expression of diverse virulence factors during infections (e.g., expression of adhesins early during colonization versus production of toxins late in infection to facilitate tissue spread) hints at the existence of multiple regulatory elements that respond to a variety of different environmental signals [41–43]. Considering the antimicrobial susceptibility patterns, the highest resistance rates were determined for cefoxitin in 53.8% and trimethoprim-sulfamethoxazole in 46.2% of the isolates. The highest frequency of *sea* gene responsible for the synthesis of the SEA was detected among strains that exhibited phenotypic resistance to chloramphenicol, erythromycin, cefoxitin and trimethoprim-sulfamethoxazole antibiotics. The *sec* gene among strains that acquired resistant to gentamicin and *sed* in strains with phenotypic resistance to doxycycline, levofloxacin, novobiocin were the most abundant identified SEs genes. Clindamycin and rifampicin resistant strains showed higher frequencies of CoNS containing the *seh* gene in association with other SEs genes. In addition, in this regard, the largest frequency of *see* gene was found among isolates showing phenotypically resistance toward cefoxitin and doxycycline antibiotics and 18% of all CoNS isolates harbored *see* gene and exhibited resistance to trimethoprim-sulfamethoxazole, clindamycin, levofloxacin and novobiocin antibiotics concurrently. Regarding the results of statistical analysis in CoNS isolates observed a significant association between the incidence of SEs genes and resistance to antimicrobial agents. Schroeder et al, and Motamedi et al. described the role of the occurrence infective determinant-associated genes in the development of resistance to the antimicrobial agents and fatal *Staphylococcal* infections [44, 45]. 11 (12%) Out of 91 isolates were found to be resistant to levofloxacin, which the most dominant detected CoNS species was *S. epidermidis* (46%), followed by *S. haemolyticus* (36%) and *S. saprophyticus* (18%). The most remarkable percentage of the resistant isolates were found in the blood infections (64%), followed by catheter-associated urinary tract infections (18%). 45% of the identified isolates that exhibited phenotypic resistance to levofloxacin were carrying the SEs and TSST_1_ genes that *seb *+ *sec* were the most frequent, and the majority of toxigenic strains of CoNS were shown to belong to *S. haemolyticus*. In this regard, our finding is similar to another study in which the proportion of resistance to levofloxacin ranged from 0% in methicillin-resistant *S. haemolyticus* (MRSH) to 7.7% in methicillin-resistant *S. epidermidis* (MRSE) strains [46]. These findings confirm which levofloxacin is a fluoroquinolone that exerts a potent effect against MRSH, MRSE and CoNS strains. It should be noted that, the access very little available literature on any information concerning the *gyrA*, *gyrB*, *grlA*, and *grlB* genes in *staphylococci* isolates was available. Among the phenotypically-resistant strains to levofloxacin, 5 (45.4%) out of 11 isolates determined to be positive for carrying of levofloxacin-conferring genes. Notably, the combinations of resistance genes were conserved in these strains. Amongst the positive isolates, 6 (54.5%) were positive for four genes, 3 (27.2%) were positive for three genes and 1 (9%) harbored two genes. In levofloxacin-resistant *S. haemolyticus* strains the *grlA *+ *grlB* and *gyrB* genes were responsible for inducing resistance to levofloxacin. In a survey carried out by Osman et al., *gyrA*, *gyrB* and *grlA* genes in *S. haemolyticus* strains were absent and 66.6% and 33.33% of MSSA (methicillin susceptible *Staphylococcus aureus*) isolates carried *gyrA* and *gyrB* genes, which in contrary to our results the prevalence percentage of these genes were reported with high incidence, 50%, 75% and 50%, respectively [1]. According to published results by Osman et al., the prevalence of *gyrA* and *grlA* genes among various *staphylococcus* species in accordance with our results were with high incidence (63% and 70.4%, respectively) and *gyrB* gene in contrary with our findings was identified with incidence low, 26% [47]. Fluoroquinolones (FQs) are categorized as effective antibiotics against a wide variety of organisms and having a role in the chemotherapy and postexposure prophylaxis for organisms, that could be used in biologic warfare. A extreme resistance could be attributed to the extensive use of FQs which can led to the development of drug resistance among *staphylococcus* isolates and other bacterial species [47]. Regarding to the prevalence proportion of insertion sequences, IS256 and IS257 were identified with the highest frequency among *S. haemolyticus* and *S. epidermidis* strains with the frequencies of 58.6%, 41.4% and 38.2%, 54.4%, respectively. Studies analyzing the prevalence rate of IS256 among *S. epidermidis* strains have been reported diverging results from 46.7% to 81% [48–50]. Previous reports have demonstrated that IS256 is significantly associated with multi-resistant, biofilm-forming *S. epidermidis* isolates resident in the hospital setting [51]. It was also indicated association of the IS256 with the genomes of aminoglycoside-resistant *staphylococci* and *enterococci* isolates [52, 53]. In addition, in this study statistically found significant association between the incidence of IS256 and resistance to gentamicin as well as between the presence of IS257 and resistance to cefoxitin (*p value* 0.033 and 0.004, respectively). The IS257 is a mobile genetic element, which associated with genes mediating biofilm formation and genes conferring resistance to beta-lactamase, aminoglycosides and tetracycline antibiotics [54, 55]. The IS256 can be used as a potential molecular marker to discriminate invasive strains from commensal strains of *S. epidermidis* [56]. Montanaro et al. demonstrated a dramatic correlation between the presence of IS256 and resistance to gentamicin [57]. Considering the antimicrobial susceptibility patterns, strains harboring IS256 and IS257 were the most frequent among those with resistance to cefoxitin (53% and 76%, respectively). It was also found that IS256 may influence the expression of pathogenesis-related genes [50]. In current research IS256 was the most common among isolates harboring *sea* also statistically found significant relationship between production of SEA and the occurrence of IS256.

### Limitations

One of the limitation of this study was evaluation of the expression level of genes responsible for production of superantigens and antibiotic resistance-inducing genes and also the sample size, if we could include higher the sample sizes in our research we would definitely get better and more reliable results.

## Conclusion

The high prevalence of SEs-encoding genes indicating a potential risk for causing human-originated food poisoning and also the emergence of multidrug-resistant bacteria is a serious problem for public health. Due to the correlation between the incidence of SAgs and the patterns of antibiotic resistance in CoNS isolates, therefore, detection of isolates harboring toxin genes associated with antibiotic resistance is rapidly becoming a significant issue of concern. The high degree of coexistence of resistance to cefoxitin and the presence of IS257 among different CoNS species indicates their role in infectious diseases. In addition, correlation between the incidence of insertion element IS256 and production of SEA enterotoxin as well as resistance toward gentamicin conferring selective advantage for pathogenesis and survival of invasive CoNS isolates in hospital settings.

## Data Availability

The datasets used and/or analyzed during the current study are available from the corresponding author on reasonable request.

## Abbreviations

CoNS: Coagulase-negative *staphylococci*
SAgs: Superantigens
SFP: *Staphylococcal* food poisoning
ETs: Exfoliative toxins
TSST-1: Toxic shock syndrome toxin 1
PTSAgs: Pyrogenic toxin superantigen family of exotoxins
QRDRs: Quinolone resistance determining regions
DAD: Disk agar diffusion
CLSI: Clinical and Laboratory Standards Institute
